# Changes in expression of PD-L1 on peripheral T cells in patients with melanoma and lung cancer treated with PD-1 inhibitors

**DOI:** 10.1038/s41598-021-93479-z

**Published:** 2021-07-28

**Authors:** Sarah J. Dart, Alistair M. Cook, Michael J. Millward, Alison M. McDonnell, Wee L. Chin, Muhammad U. Hakeem, Tarek M. Meniawy, Samantha E. Bowyer

**Affiliations:** 1 Faculty of Health and Medical Sciences, The University of Western Australia, Perth, WA Australia; 2 National Centre for Asbestos Related Diseases, Perth, WA Australia; 3 Department of Medical Oncology, Sir Charles Gairdner Hospital, Perth, WA Australia; 4 Institute for Respiratory Health, Nedlands, WA 6009 Australia

**Keywords:** Tumour biomarkers, Skin cancer, Melanoma, Cancer, Lung cancer, Non-small-cell lung cancer

## Abstract

Advances in cancer immunology have increased the use of immune checkpoint inhibitors in clinical practice, however not all patients respond, and treatment can have severe side-effects. Blood-based immunological biomarkers are an attractive method for predicting which patients will respond to therapy, however, reliable biomarkers for immune checkpoint blockade are lacking. This study aimed to identify patients before or early in treatment who would best respond to PD-1 inhibitors. We hypothesised that higher baseline PD-L1 and/or PD-1 on peripheral blood T cells could predict radiological response to PD-1 inhibitors. This pilot prospective cohort study assessed 26 patients with melanoma or non-small cell lung cancer, treated with pembrolizumab, nivolumab, or nivolumab/ipilimumab combined. Response was assessed by RECIST 1.1. Peripheral blood lymphocytes collected at baseline, after one cycle, 10 weeks and at discontinuation of therapy were analysed by flow cytometry. Patients with a higher proportion of PD-L1^+^ T cells at baseline had improved objective response to PD-1 inhibitor therapy, and patients with a lower proportion of regulatory T cells at baseline experienced more immune-related adverse events. These findings may prove useful to assist in clinical decision making. Further studies with larger cohorts are required to validate these findings.

## Introduction

Checkpoint immunotherapy is the most important advance in cancer treatment for several decades. It is a rapidly evolving field with worldwide approvals in many tumour types, with malignant melanoma and non-small cell lung cancer (NSCLC) being at the forefront of drug development. However, there remains an unmet need for reliable biomarkers of response to immune checkpoint inhibitors.

Programmed-cell death receptor-1 (PD-1) inhibitors can cause significant and durable anti-tumour responses with manageable toxicity profiles. A number of phase III studies have established the superiority of single agent PD-1 inhibitors to either chemotherapy or the cytotoxic T-lymphocyte-associated protein-4 (CTLA-4) inhibitor ipilimumab in the first and subsequent line treatment of melanoma^1–4^. Combination of nivolumab, a PD-1 inhibitor, with ipilimumab has been proven in phase III trials to have superior efficacy in terms of response and survival over ipilimumab alone in melanoma, but with more significant toxicity^5^. In advanced NSCLC, single agent PD-1 inhibitors demonstrated superior efficacy to chemotherapy in second and subsequent lines of treatment in both squamous and non-squamous histologies^6–8^. These agents have achieved regulatory approvals as first line treatment either as monotherapy or in combination with chemotherapy^9–11^. However, these therapies are both expensive and potentially toxic. As such, biomarkers are needed to rationalise the use of PD-1 inhibitors and identify patients who may potentially benefit from combinatorial approaches rather than single agent treatment.

Immunological parameters in the tumour microenvironment can be predictive of response to PD-1 inhibitors, with the most commonly reported predictive biomarker being PD ligand-1 (PD-L1) expression in tumour tissue^12,13^. In some tumour types, PD-L1 up-regulation on tumour and/or tumour-infiltrating immune cells are mechanisms by which tumours evade the host immune response^14^. To date, PD-L1, assessed using immunohistochemistry on tumour tissue collected prior to therapy, has been reported as a predictive biomarker of response to PD-1 inhibitors in melanoma and lung cancer^13,15^. Treatment with pembrolizumab, another PD-1 inhibitor, is associated with significantly longer progression-free and overall survival compared with platinum doublet chemotherapy in the first line setting for patients with advanced NSCLC expressing PD-L1 on at least 50% of tumour cells^10^. However, patients with lower values can still benefit from therapy, and vice versa^13,15^ and the dynamic and heterogeneous nature of an adaptive immune response limits PD-L1 as a standalone biomarker^16^. These assays can also be somewhat subjective as they require the interpretation of a pathologist. Biopsies also present a number of drawbacks: they are an inherently invasive procedure, the same area by definition cannot be sampled sequentially, and in any case may not be wholly representative for reasons of tumour heterogeneity^17,18^, either within the same tumour, or between multiple tumours in a single patient.

Tumour infiltrating lymphocytes, T cell receptor repertoire, immune gene signatures, and neoantigen burden have all been explored as potential biomarkers but are insufficient in isolation to be clinically relevant in ruling in or out the use of checkpoint inhibitors^19^. There is evidence that immunological biomarkers found in peripheral blood can reflect the immunological milieu of the tumour microenvironment^15,20,21^. In a study by Iwahori et al.^21^, peripheral T cell cytotoxicity was shown to be correlated to T cell function in NSCLC tumours when compared to normal lung tissue, including the expression of T-cell immunoglobulin and mucin-domain containing-3 (TIM-3) by CD8^+^ T cells, and the ability of peripheral blood mononuclear cells (PBMC) to produce interferon gamma. Huang et al.^20^ also showed concordance between the presence of specific T cell clones in blood and tumour, and that Ki67^+^HLA-DR^+^CD38^+^ CD8^+^ T cells in the blood reinvigorated by anti-PD-1 therapy also had counterparts in the tumour.

Blood-based biomarkers are ideal as they are dynamic and can potentially reflect treatment effects in real time as well as being easily accessible. However, despite the advantages provided by working with PBMC, reliable cellular biomarkers in this compartment are still somewhat lacking and require further investigation. Here, we investigated immune biomarkers in peripheral blood before and during PD-1 inhibitor therapy as a predictor of response in a small cohort of patients with advanced melanoma or NSCLC. We hypothesised that higher pre-treatment PD-1 and PD-L1 expression on peripheral blood T cells would be predictive of objective radiological response to PD-1 inhibitor therapy. In addition, we hypothesised that treatment with a PD-1 inhibitor would decrease PD-1 and PD-L1 expression on T cells as well as increase proportions of proliferating (Ki67^+^) and activated (ICOS^+^) CD8^+^ T cells.

## Results

### Patient characteristics

**Figure 1:**
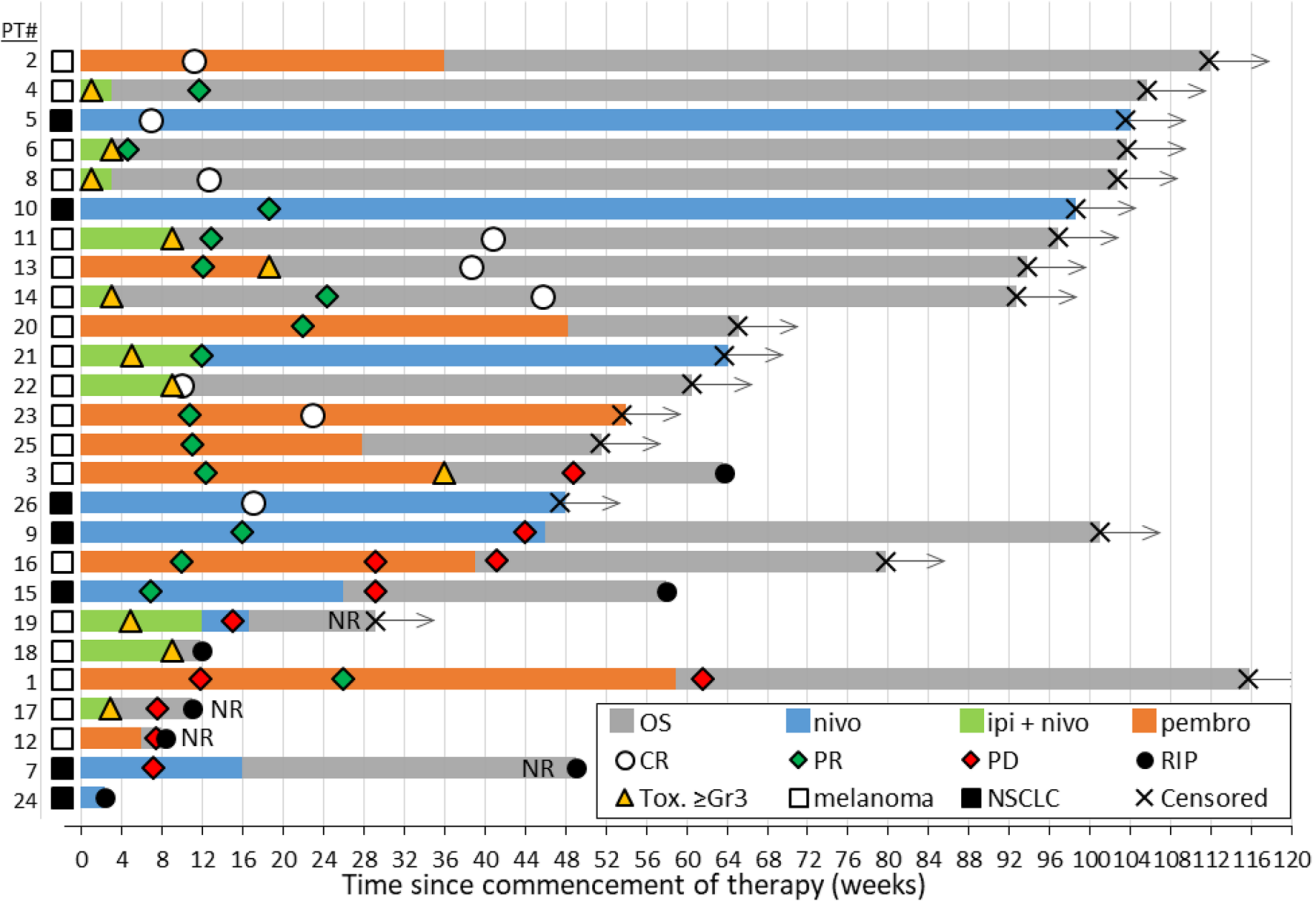
Swimmer plot showing individual patient characteristics with respect to time since commencement of therapy including: treatment type and duration, overall survival (OS), date of death (RIP) or censorship date, and timing of radiological responses of either complete response (CR), partial response (PR) or progressive disease (PD). Timing of toxicities of grade 3 or above are also noted. Patients are ordered time until either first recorded disease progression or time until censorship. *NR* non-responding patient. Patients 24 and 18 were not assessed for radiological response.

Twenty-six patients were screened and enrolled in this study between February 2016 and May 2017. The median age was 67.5 years and 73% were male (Table 1). Nineteen patients had melanoma and seven had NSCLC. All seven lung cancer patients were treated with single-agent nivolumab on a two-weekly basis in the second line setting. Sixteen (84%) melanoma patients received immunotherapy as their first line of treatment; 10 (53%) received combination therapy with nivolumab plus ipilimumab three weekly for 4 doses followed by nivolumab monotherapy two weekly at standard dosing, and the remaining nine patients (47%) received monotherapy with pembrolizumab administered three-weekly (Fig. 1).

**Table 1:** Patient characteristics.

	All patients (n = 26) n (%)	Melanoma (n = 19) n (%)	NSCLC (n = 7) n (%)
**Age (years)**			
Median	67.5	71	64
**Sex**			
Male	19 (73)	15 (79)	4 (57)
Female	7 (27)	4 (21)	3 (43)
**Prior therapy**			
Treatment naive	16 (62)	16 (84)	0 (0)
≥ 1	10 (38)	3 (16)	7 (100)
**Histopathology**			
Melanoma (BRAF mutation negative)		16 (84)	
Melanoma (BRAF activating mutation)		3 (16)	
NSCLC (adenocarcinoma)			4 (57)
NSCLC (SCC)			3 (43)
**Immunotherapy**			
Pembrolizumab	9 (35)	9 (47)	0 (0)
Nivolumab	7 (27)	0 (0)	7 (100)
Ipilimumab plus nivolumab	10 (38)	10 (53)	0 (0)
**Stage of disease (AJCC)**			
Recurrent stage III	3 (12)	0 (0)	3 (43)
Stage IV	23 (88)	19 (100)	4 (57)

Patients were censored as of May 1st, 2018; 1 year following recruitment of the final patient. As of this date, seven patients were deceased with five of these deaths due to progressive cancer. One patient (#19) was lost to follow-up following progression. Four patients (15%) were classified as non-responders (PD), the response of two patients was unknown, and the remainder were classified as responders (CR/PR). No patients were classified as responders with stable disease (SD). Six patients were alive after radiological progression of disease, five of these patients having demonstrated an initial partial response to therapy followed by progression. Median progression free survival (PFS) for all patients was 11.6 months, whilst median overall survival (OS) was 14.9 months (Table 2).

**Table 2:** Response assessment and survival outcomes.

	All patients (n = 26) n (%)	Melanoma (n = 19) n (%)	NSCLC (n = 7) n (%)
ORR	76.9	78.9	71.4
**Best response**			
PD	4 (15)	3 (16)	1 (14)
SD	0 (0)	0 (0)	0 (0)
PR	11 (42)	8 (42)	3 (43)
CR	9 (35)	7 (37)	2 (29)
Unknown	2 (8)	1 (5)	1 (14)
**Toxicity**			
≥ Grade 3	12 (46)	12 (46)	0 (0)
Steroid requirement	11 (42)	11 (42)	0 (0)
Median time to toxicity (days)	61.5	61.5	NA
**Discontinuation (n = 21)**			
Death/PD	9 (35)	5 (5)	4 (57)
Toxicity	8 (31)	8 (42)	0 (0)
Ongoing	3 (11)	3 (16)	0 (0)
**CR/PR**			
Progression after response	6 (23)	3 (16)	3 (43)
6 month PFS rate	18 (69)	14 (74)	4 (57)
1 year PFS rate	14 (54)	11 (58)	1 (14)
Progression free survival—median, months (range)	11.6 (8.3–14.8)	14.0 (10.1–17.8)	6.7 (0.9–12.6)
6 month OS rate	22 (85)	16 (84)	6 (86)
1 year OS rate	18 (69)	14 (74)	4 (57)
Overall survival—median, months (range)	14.9 (11.9–17.8)	14.7 (11.2–18.1)	13.4 (6.9–19.8)

There were grade 3 or higher immune-related adverse events (irAE) in 12 patients (46%), all within the melanoma cohort. Ten patients experiencing significant toxicity had received ipilimumab in combination with nivolumab, with the remaining two receiving pembrolizumab monotherapy. Of these 12, all except one patient required systemic corticosteroids as part of their treatment. One patient (#18) did not receive steroids due to rapid deterioration and death due to toxicity. Median time to onset of grade 3 immune related events was 61.5 days (range 19–265 days).

### Sample collection

Of 26 patients enrolled, all patients had baseline samples collected and 25 had a subsequent sample collected at day 21. No patients received steroids prior to baseline or day 21 samples. Nineteen patients had a week 10 sample taken and 14 patients had a further sample collected on progression or discontinuation of therapy. Two of the 11 patients requiring steroids received steroids prior to their week 10 sample collection, and three prior to their final sample collection.

### Baseline T cell PD-L1 expression is associated with response

**Figure 2:**
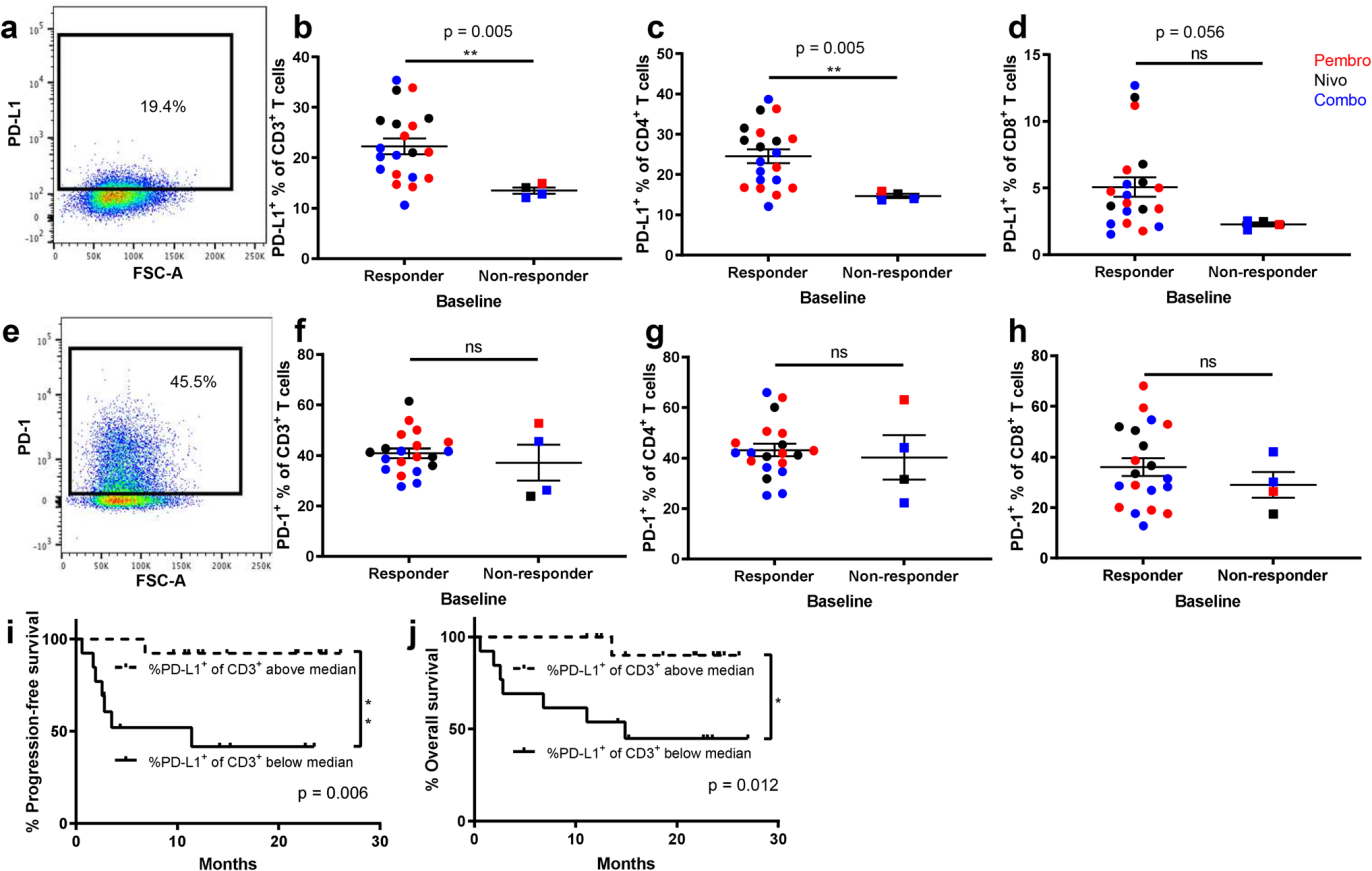
Patients with a higher baseline PD-L1 expression on T cells have a more favourable overall best response. PBMC collected from patients were assessed for PD-L1 and PD-1 expression in the total CD3^+^, CD3^+^CD4^+^ and CD3^+^CD8^+^ lymphocyte populations by flow cytometry, using fluorescence-minus one controls to set gates. Frequencies of PD-L1 (**a–d**) and PD-1 (**e–h**) in each population for total responder (n = 20) and non-responder patients (n = 4) were compared using a Mann–Whitney test; **p ≤ 0.005; error bars, SEM. Data is displayed with treatment groups colour-coded; pembrolizumab, red; nivolumab, black; combination nivolumab/ipilimumab, blue. Kaplan–Meier plot showing progression-free survival (**i**) and overall survival (**j**) of patients with CD3^+^ T cell %PD-L1 expression above and below the median, p values calculated using Mantel-Cox Log-rank test. See also Supplementary Fig. S2.

The primary aim of this study was to examine whether the proportion of peripheral blood T cells expressing PD-L1 prior to treatment with PD-1 inhibitors was associated with better objective response to therapy. Following flow cytometry analysis of patient PBMC (Supplementary Fig. S1), we found that patients responding to treatment had a higher frequency of PD-L1 expression on CD3^+^ T cells than those who did not respond (p = 0.005, Fig. 2a,b). This was also the case for CD3^+^CD4^+^ T cells (p = 0.005, Fig. 2c). Within the PD-L1^+^ populations of CD3^+^ and CD3^+^CD4^+^ T cells, the median fluorescent intensity (MFI) of PD-L1 between responders and non-responders trended higher in responders vs. non-responders, but did not reach statistical significance (CD3, p = 0.07; CD4, p = 0.1; Supplementary Fig. S2). Whilst the frequency of PD-L1^+^ expression in CD3^+^CD8^+^ T cells (Fig. 2d) also trended higher in responders vs. non-responders, it did not reach statistical significance (p = 0.06). We found no significant difference in the PD-1^+^ proportion or MFI of cells at baseline within total (p = 0.7), CD4^+^ (p = 0.6), or CD8^+^ (p = 0.4) T cells between responders and non-responders (Fig. 2e–h, Supplementary Fig. S2). None of these described findings were treatment group specific. We had initially hypothesised that a cut-off of </> 1% of T cells expressing PD-1 or PD-L1 at baseline may be predictive. However, our results did not demonstrate values within this low range. The lowest value encountered with our cohort of patients was approximately 10%. The median in this study was 20.2%, and we therefore adopted this data-driven cut-off of above and below the median for our analysis. When patients were grouped according to PD-L1 expression above and below the median, significant differences were observed in favour of those patients with higher PD-L1 expression on T cells for both PFS and OS (Fig. 2i,j). In this cohort, six patients progressed on anti-PD-1 therapy after an initial response.

### PD-1 inhibitors stimulate changes in immunological parameters after one cycle of treatment

**Figure 3:**
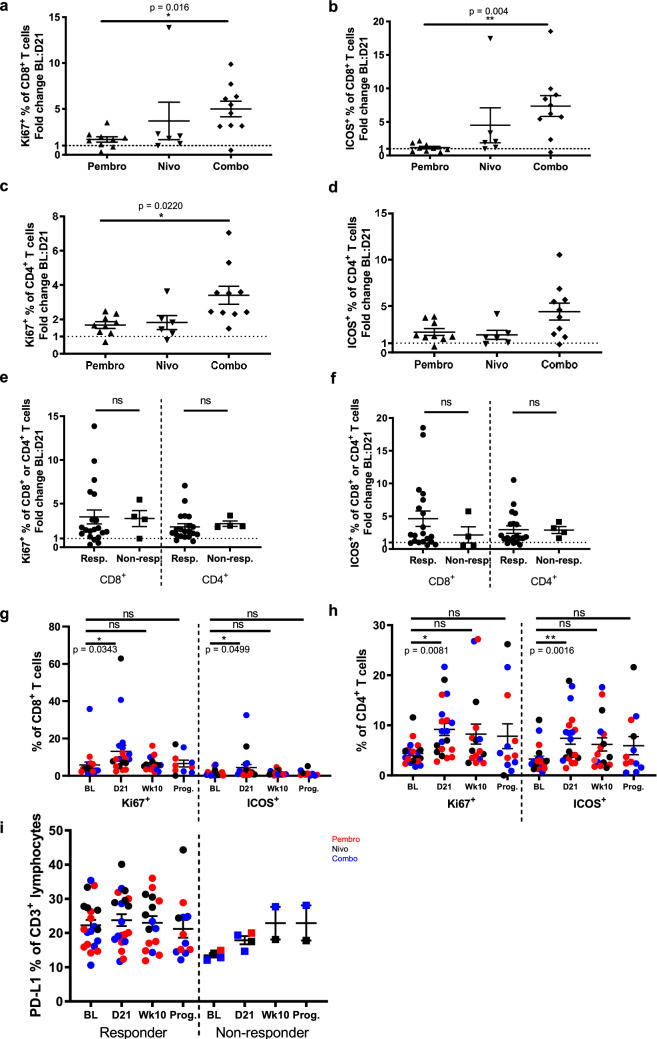
Increased activation and proliferation of CD8^+^ and CD4^+^ lymphocytes after therapy. Flow cytometry analysis of CD8^+^ and CD4^+^ lymphocytes collected from all patients. Fold change in the proportion of Ki67^+^ (**a**) and ICOS^+^ (**b**) CD8^+^ T cells, and Ki67^+^ (**c**) and ICOS^+^ (**d**) CD4^+^ T cells between baseline and day 21, comparing patients treated with pembrolizumab (pembro; n = 9), nivolumab (nivo; n = 6), or combination nivolumab/ipilimumab (combo; n = 10). Fold change in the proportion of (**e**) Ki67^+^ and (**f**) ICOS^+^ CD8^+^ and CD4^+^ T cells between baseline and day 21, in responding (n = 20) vs non-responding (n = 4) patients. Proportion of CD8^+^ (**g**) and CD4^+^ (**h**) lymphocytes expressing Ki67 and ICOS at baseline (BL; n = 20), day 21 (D21; n = 20), week 10 (Wk10; n = 18) and time of discontinuation or progression (Prog.; n = 10), for responding patients only, colour-coded by treatment group (**i**) Proportion of CD3^+^ lymphocytes expressing PD-L1 at baseline (BL), day 21 (D21), week 10 (Wk10) and time of discontinuation or progression (Prog.), for responders (n = 20, 20, 18, 10) and non-responders (n = 4, 4, 2, 2), colour-coded by treatment group. p values calculated by Kruskal–Wallis test with multiple comparisons (**a–d**), Mann–Whitney test (**e,f**) or mixed effects analysis (**g–i**); *p ≤ 0.05, ** p ≤ 0.005; error bars, SEM.

To assess changes in immunological parameters in response to PD-1 inhibitor therapies, we compared patient PBMC collected at baseline and after one cycle of treatment (day 21). We demonstrated that treatment with immune checkpoint inhibitors increased the proportions of proliferating (Ki67^+^, Fig. 3a,c) and activated (ICOS^+^, Fig. 3b,d) CD8^+^ and CD4^+^ T cells, predominantly in patients treated with combination nivolumab/ipilimumab. However, this did not correlate with clinical outcomes (Ki67, p = 0.6; ICOS, p = 0.2; Fig. 3e,f). Proportions of activated and proliferating CD8^+^ and CD4^+^ T cells returned to baseline levels by week 10 of treatment (Fig. 3g,h). Importantly, treatment with a PD-1 inhibitor did not decrease PD-L1 expression on T cells as hypothesised (Fig. 3i). Interestingly, unlike responders, all non-responding patients demonstrated a slight increase in PD-L1 expression on CD3^+^ T cells which continued until week 10, although due to the small sample size this difference was not statistically significant (baseline to day 21, p = 0.1).

### Low proportion of regulatory T cells at baseline is associated with toxicity after treatment with PD-1 inhibitors

**Figure 4:**
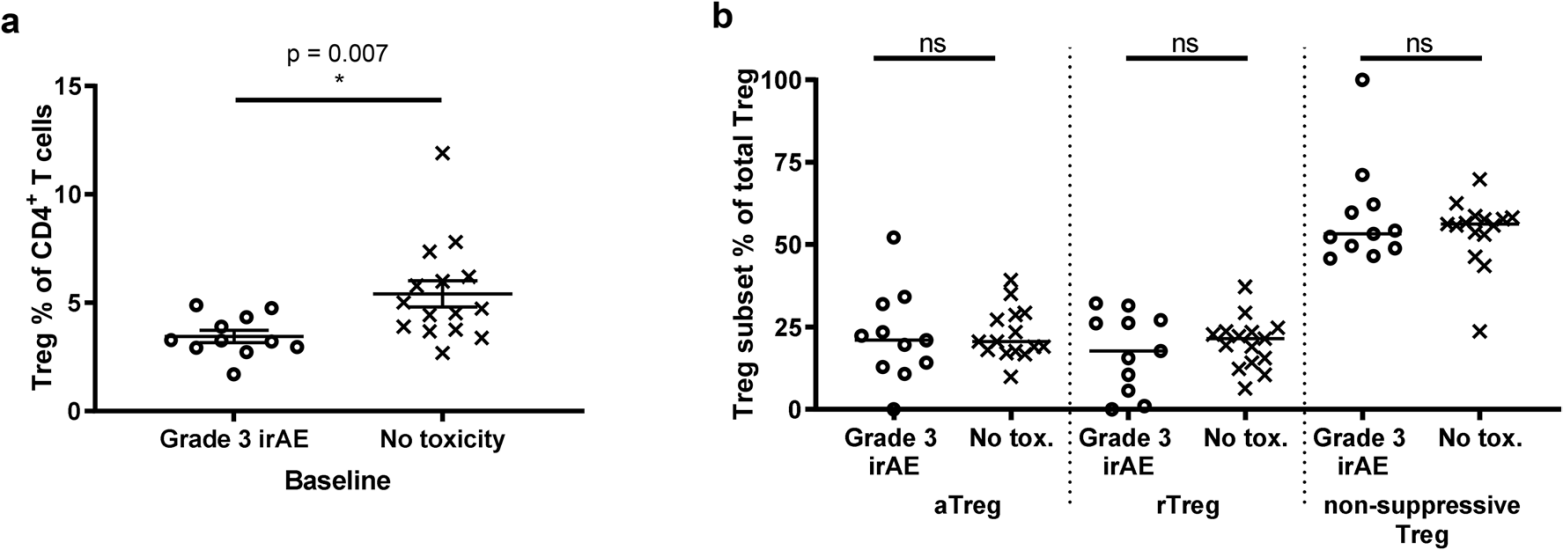
Patients who go on to have grade 3 irAEs have lower proportions of total Tregs at baseline. (**a**) Baseline Treg (CD25^+^CD127^lo^FoxP3^+^) proportion of CD3^+^CD4^+^ lymphocytes in patients experiencing grade 3 irAE (n = 11) or no toxicity (n = 15). (**b**) Frequencies of Treg subsets at baseline: CD45RA^-^FoxP3^hi^ activated Treg (aTreg); CD45RA^+^FoxP3^lo^ resting Treg (rTreg); CD45RA^-^FoxP3^lo^ non-suppressive Treg cells. Mann–Whitney test; *p ≤ 0.05; error bars, SEM.

In our cohort, all patients treated with combination nivolumab/ipilimumab therapy, plus two treated with pembrolizumab alone, exhibited irAEs of grade 3 or above. We analysed patient PBMCs collected at baseline by flow cytometry for total Treg cells and Treg sub-populations, as identified by Miyara, Yoshioka^22^ (Supplementary Fig. S1). We determined that those patients who experienced an irAE of grade 3 or higher had a significantly lower mean overall Treg (CD4^+^CD25^+^CD127^lo^FoxP3^+^) proportion of total CD4^+^ T cells at baseline (p = 0.007, Fig. 4a). We subsequently examined the activated (aTreg; CD45RA^−^FoxP3^hi^), resting (rTreg; CD45RA^+^FoxP3^lo^) and non-suppressive (CD45RA^−^FoxP3^lo^) Treg sub-populations, however none of these were found to contribute more than the others toward the elevated Treg level in the group who would not go on to experience irAEs of grade 3 or above (p = 0.9, p = 0.8 and p = 0.9 respectively; Fig. 4b).

## Discussion

Immune checkpoint inhibitors are now widely used in the clinic, with approvals in a rapidly increasing variety of malignancies. However, the search for predictive or prognostic biomarkers is ongoing^23–25^. In this prospective pilot study, we found that a higher pre-treatment proportion of peripheral blood T cells expressing PD-L1 was associated with improved response. Expected levels of PD-L1 on peripheral T cells were not well-defined in the literature prior to this study’s design, with ranges of > 0–3.6%^26,27^. The median in this study was 20.2%, and was adopted as a data-driven cut-off of above and below the median for our analysis. In contrast to PD-L1, baseline PD-1 expression on peripheral T cells in our cohort did not correlate with objective response rates.

High expression of PD-L1 is a negative prognostic marker in both melanoma and lung cancer and is widely regarded as a potential mechanism of immune escape^26,28,29^. PD-L1 expression by T cells as a receptor for B7-1 has previously been reported, however, the signalling mechanisms and immunological effects of PD-L1 expression on T cells have yet to be described in depth^30,31^. A prospective study by Arrieta et al.^26^ demonstrated that survival among lung cancer patients with a higher percentage of PD-1^+^ and PD-L1/2^+^ CD3^+^ T cells was reduced. All patients were treatment naïve, however no comment was provided as to what subsequent treatments these patients received^26^. Meniawy et al.^29^ also report that lung cancer patients with a higher PD-L1^+^ proportion of T cells a week following epidermal growth factor receptor tyrosine kinase inhibitor (EGFR TKI) treatment were more likely to progress (OR 30.3, p < 0.01) and had shorter PFS (1.6 vs. 8.8 months; p < 0.01) and OS.

In our cohort, a higher percentage of PD-L1 expression on CD3^+^ T cells correlated with more favourable response to immune checkpoint inhibition as well as improved progression free and overall survival. This suggests that although this may be a negative prognostic factor in the pre-immunotherapy era, it may be transformed into a positive predictive factor for response to PD-1 pathway inhibitors and survival. The most straightforward explanation for this observation is that for patients with more PD-L1-expressing T cells, the PD-1 pathway may be a mechanism of immune escape by these tumours, and therefore these patients would benefit from blocking the PD-1/PD-L1 receptor/ligand interaction. It may be that T cell PD-L1 expression levels can provide important information in the decision-making process concerning treatment options, e.g. patients who have high T cell PD-L1 at baseline may respond better to immunotherapy as a monotherapy but patients with low values may benefit from combination approaches with other immunotherapy agents, targeted therapy or chemotherapy. Further examination in a larger cohort is therefore warranted, and in future could assist in setting a guideline threshold value for T cell PD-L1 expression levels above or below which therapeutic decisions can be made.

Changes in immunological parameters in response to PD-1 inhibitor therapies, including increased proportions of proliferating and activated CD8^+^ and CD4^+^ T cells, were most notable in patients treated with nivolumab plus ipilimumab, after one cycle of treatment. However, increased proportions of proliferating and activated CD8^+^ and CD4^+^ T cells did not correlate with radiological response as hypothesised. This supports the findings of Huang et al.^20^ demonstrating that an immunologically on-target effect can be seen with PD-1 inhibitor treatment; but the effect on T cell activation and proliferation is not necessarily clinically significant and can depend on additional factors such as tumour burden.

With respect to PD-1 expression, however, it is also possible that Huang et al.^20^ may have underestimated frequencies of PD-1 staining at 21 days post-therapy, since in our hands pembrolizumab binding was seen to have greatly reduced by this time point. Their detection method solely employed indirect detection using a secondary antibody against bound pembrolizumab, whereas we used both an anti-human IgG_4_ to detect bound therapeutic anti-PD-1^20,32,33^ plus a primary anti-PD-1 detection antibody in the same assay, to allow the detection of unbound PD-1 molecules. The differences we observed between nivolumab- and pembrolizumab-treated patients (namely that anti-human IgG_4_ alone is insufficient to reflect the frequency of PD-1 expression in patients receiving pembrolizumab at day 21) serve as a timely reminder of the pharmacokinetic differences between ‘similar’ drugs from different manufacturers. Thus, several factors need to be carefully considered prior to using such assays, including the particular antibody clones used for detection, specific assay conditions including buffers and staining protocols, the time elapsed between a patient receiving treatment and the detection assay being performed, and differences in treatment regimens between drugs (e.g. 3-weekly for pembrolizumab vs 2-weekly for nivolumab).

A recent study by Kim et al.^34^ examined responses to pembrolizumab treatment in a small cohort of NSCLC patients. They demonstrated that a greater fold increase in Ki67^+^ expression between PD-1^+^CD8^+^ PBMC collected immediately before treatment versus 7 days after the first dose was associated with durable clinical benefit. Whilst our study did not examine PBMCs within such a short timeframe, we still detected an increase in Ki67 expression 21 days after the first dose of PD-1 inhibitors, albeit in the wider CD8^+^ T cell population rather than focussing on the PD-1^+^CD8^+^ subpopulation. Kim et al.^34^ did not report on a significant change in Ki67 expression between day 0 and day 21, although this data was from only nine patients and thus may have masked any real differences through a lack of statistical power.

In a phase I trial of the anti-PD-L1 antibody atezolizumab, the CD8^+^HLA-DR^+^Ki67^+^ activated T cell subpopulation had increased by day 21, although this did not correlate with response or progression^35^. Preliminary findings from the Checkmate 069 study, presented at ASCO in 2016 by Postow et al.^36^, found there was a greater increase in Ki67 expression by CD8^+^ T cells in patients treated with ipilimumab plus nivolumab compared to ipilimumab alone at day 21 compared to day 0—however differences between combination therapy and nivolumab alone were not reported. Here, our data show greater proliferation in those patients receiving combination CTLA-4/PD-1 inhibitor therapy when compared to PD-1 inhibitor alone. Taken together, these findings suggest that both PD-1 and CTLA-4 inhibitors exert a proliferative effect on CD8^+^ T cells as monotherapies, and that these effects appear additive or synergistic when used in combination—as might be expected. Our observation that CD4^+^ T cells also display elevated activation (as measured by Ki67 and ICOS) in combined CTLA-4/PD-1 inhibitor therapy versus PD-1 inhibitor monotherapy is consistent with other reports correlating CTLA-4 inhibitor therapy to the expansion of the ICOS^+^CD4^+^ T cell population^37–39^.

We evaluated whether immunological parameters at baseline could be predictive of irAEs and whether occurrence of irAEs following treatment was associated with clinical outcome. Our study cohort was enriched with patients experiencing toxicity of CTCAE grade 3 or above, notably within the patients treated with combination ipilimumab and nivolumab; 100% of combination therapy patients here compared with an expected rate of approximately half^4^. We determined that a lower proportion of total Tregs within the CD4^+^ T cell population (CD4^+^CD25^+^CD127^lo^FoxP3^+^) was associated with increased toxicity. As toxicity can be severe, this finding could be clinically relevant since patients with low baseline Tregs may warrant closer monitoring for irAEs. Since all but two patients in our study with severe irAEs were deemed to be responders, we did not have statistical power to test whether occurrence of irAEs correlated with response. Confirmation of Treg populations in patients not experiencing immune related adverse events would be needed to validate this finding in the context of dual checkpoint blockade, which was not possible within the confines of this study.

In summary, higher pre-treatment frequency of PD-L1 expression on peripheral blood T cells was associated with improved response to PD-1 inhibitors and may prove a useful parameter to assist in clinical decision making. Furthermore, a lower proportion of regulatory T cells at baseline was associated with increased likelihood of on-treatment high grade toxicity (Grade 3–5) and could help distinguish patients who require closer monitoring for irAEs. We would highlight the preliminary nature of this small cohort study, and recommend that these findings should be considered hypothesis-generating; confirmation of these findings by a larger study will be required. In addition, interpretation may be complicated by the inclusion of two cancer types and two PD-1 inhibitors, plus the fact that a number of patients additionally received a CTLA-4 inhibitor, radiation and/or steroids. Notwithstanding, our findings imply a wider relevance across a broad spectrum of clinical settings if replicated in future studies.

## Methods

### Patient eligibility

Patients with advanced melanoma or NSCLC prospectively commencing a PD-1 inhibitor as part of standard care, either as a monotherapy or in combination with ipilimumab, were eligible to participate. All patients provided written informed consent, and the study was approved by the Sir Charles Gairdner Hospital Human Research Ethics Committee (Study No: 2015-178) and conducted in accordance with the Declaration of Helsinki.

### Study design

This was a pilot prospective cohort study conducted at a single centre in Western Australia. The primary endpoint was objective tumour response rate (ORR) according to the proportion (</> 1%) of PD-L1 expressing CD3^+^ T cells at baseline. Secondary endpoints were time to event outcomes, changes in T cell proliferation markers as well as other immune cell correlates.

### Assessment of response

Patients underwent a Computer Tomography (CT) scan ± a fluorodeoxyglucose positron emission tomography (FDG-PET) scan at baseline and after 8–12 weeks of treatment to assess initial response. Response was assessed as per Response Evaluation Criteria in Solid Tumours (RECIST) version 1.1. Subsequent imaging requirements were at the discretion of the treating clinician. For analysis, patients were grouped in terms of outcomes according to best response achieved; the ‘responders’ group included those who achieved either complete response (CR), partial response (PR) or stable disease (SD); ‘non-responders’ had progressive disease (PD).

### Time to event outcomes

Survival endpoints were defined as the time from commencing the PD-1 inhibitor until progression for Progression Free Survival (PFS), or death for Overall Survival (OS). Survival data were censored on the May 1st, 2018 or date of last visit if lost to follow-up, or date of death if they died of other causes. Toxicity was recorded as per the National Cancer Institute Common Toxicity Criteria for Adverse Events (CTCAE) Version 4.03.

### Peripheral blood mononuclear cell (PBMC) isolation

Peripheral blood samples were collected within 48 h prior to commencement of therapy, then at day 21, week 10, and on discontinuation of therapy or disease progression. Blood was collected in BD Vacutainer Cell Preparation Tubes with sodium heparin anti-coagulant and mixed by inversion. Tubes were spun for 30 min at room temperature at 1500 rcf, brake off, to isolate mononuclear cells. Cells were washed twice in phosphate buffered saline (PBS), counted and resuspended at 1–4 × 10^6^ mL^−1^ in Roswell Park Memorial Institute Medium (RPMI 1640), supplemented with 10% foetal calf serum (FCS), 20 mM HEPES (4-(2-hydroxyethyl)-1-piperazineethanesulfonic acid) and 10% dimethylsulfoxide (DMSO) for freezing. After 24 h at − 80 °C, cells were transferred to liquid nitrogen until analysis.

### Flow cytometry

Cryopreserved PBMC were thawed at 37 °C, then washed in RPMI 1640 supplemented with 10% FCS and HEPES to remove DMSO. 5 × 10^5^ cells were stained for each patient at each time-point. Cells were stained with Fixable Viability Dye ef780 (ThermoFisher), plus antibodies against CD3-BV510 (BD, UCHT1, 563109), CD4-BV711 (BioLegend, OKT4, 317440), CD8a-eVolve605 (ThermoFisher, RPA-T8, 83–0088-42), CD14-APC-eF780 (ThermoFisher, 61D3, 47-0149-42), CD19-APC-eF780 (ThermoFisher, HIB19, 47-0199-), CD25-BV421 (BD, M-A251, 562442), CD45RA-BV785 (BioLegend, HI100, 304140), CD127-PE-CF594 (BD, HIL-7R-M21, 562397), PD-1-PECy7 (BioLegend, EH12.2H7, 329918), PD-L1-BUV395 (BD, HI30, 740320), ICOS-PerCP-eF710 (ThermoFisher, ISA-3, 46-9948-42). Cells were permeabilised with FoxP3 Fixation/Permeabilisation Kit (ThermoFisher), and stained for FoxP3-PE (ThermoFisher, 263A/E7, 12-4777-42) and Ki67-FITC (BD, B56, 51-36524X). Fixation was performed with Stabilising Fixative (BD) diluted in water. Data was acquired on a BD SORP Fortessa (BD) and analysed (Supplementary Fig. S1) with FlowJo software^40^ (BD). Fluorescence minus one controls were used to determine gates.

### PD-1 occupancy assay

Given that both flow cytometry detection antibodies and the therapeutic PD-1 inhibitors bind to PD-1 cellular receptor molecules, we hypothesised that using only direct anti-PD-1 flow cytometry antibodies would result in an underestimation of PD-1 expression. To accurately quantify the expression of PD-1 on immune cells post-therapy, we adapted a protocol^20,32,33^ to identify both unoccupied and drug-bound PD-1 molecules by flow cytometry. Samples were stained in triplicate with Fixable Viability Dye plus antibodies against CD3, CD4, CD8, CD14 and CD19 as described above. PD-1 was stained either directly using anti-PD-1 PECy7, indirectly via an anti-IgG_4_, or with both methods combined (Supplementary Fig. S3). Briefly, indirect staining was achieved by incubating cells with a saturating concentration of biotinylated anti-human IgG_4_ Fc antibody (ThermoFisher, HP6025, A10663), followed by secondary staining with streptavidin PECy7 antibody (BD, 557598). Untreated control PBMC from healthy volunteers were used to confirm binding specificity and confirm that nivolumab and pembrolizumab binding and assessment were comparable (Supplementary Fig. S3). Healthy volunteer PBMCs were incubated at 4 °C with or without a saturating concentration of nivolumab or pembrolizumab for 30 min prior to PD-1 detection. By using both detection methods alone and in combination, we collected data on the expression of drug-free PD-1, bound PD-1, and total PD-1. With our direct detection antibody, there was a tendency to underestimate the percentage of PD-1^+^ cells post-therapy in patients treated with nivolumab, whilst in patients treated with pembrolizumab this was not the case (Supplementary Fig. S3).

### Statistical analyses

The relationship of PD-1 and PD-L1 expression on peripheral T cells and response to PD-1 inhibitor therapy is unknown. A sample size of 14 patients was required to give 80% power to detect a difference of 30% in objective response rates between patients with an empirical cut-off of </> 1% of CD3^+^ T cells expressing PD-L1 with a one-sided alpha of 0.1.

The Kaplan–Meier method was used to analyse these variables with relation to survival outcomes using the log-rank test. The analyses were performed in R (version 3.61) with the “survival” package (version 3.1-11)^41,42^.

Data on proportions of immune cells were correlated against either sample collection time point, radiological outcomes, or toxicity using the Student’s t test (Mann–Whitney), Kruskal–Wallis test, mixed effects test or the Wilcoxon signed-rank test. Analyses were performed using GraphPad Prism 8.0.1.

## Supplementary Information

Supplementary Information.

## Data Availability

The datasets generated and/or analysed during the current study are available from the corresponding author on reasonable request.

## References

[1] Larkin J (2015). Combined nivolumab and ipilimumab or monotherapy in untreated melanoma. N. Engl. J. Med..

[2] Ribas A (2015). Pembrolizumab versus investigator-choice chemotherapy for ipilimumab-refractory melanoma (KEYNOTE-002): A randomised, controlled, phase 2 trial. Lancet Oncol..

[3] Robert C (2015). Nivolumab in previously untreated melanoma without BRAF mutation. N. Engl. J. Med..

[4] Robert C (2015). Pembrolizumab versus ipilimumab in advanced melanoma. N. Engl. J. Med..

[5] Wolchok JD (2017). Overall survival with combined nivolumab and ipilimumab in advanced melanoma. N. Engl. J. Med..

[6] Brahmer J (2015). Nivolumab versus docetaxel in advanced squamous-cell non-small-cell lung cancer. N. Engl. J. Med..

[7] Herbst RS (2016). Pembrolizumab versus docetaxel for previously treated, PD-L1-positive, advanced non-small-cell lung cancer (KEYNOTE-010): A randomised controlled trial. Lancet.

[8] Rittmeyer A (2017). Atezolizumab versus docetaxel in patients with previously treated non-small-cell lung cancer (OAK): A phase 3, open-label, multicentre randomised controlled trial. Lancet.

[9] Gandhi L (2018). Pembrolizumab plus chemotherapy in metastatic non-small-cell lung cancer. N. Engl. J. Med..

[10] Reck M (2016). Pembrolizumab versus chemotherapy for PD-L1-positive non-small-cell lung cancer. N. Engl. J. Med..

[11] Socinski MA (2018). Atezolizumab for first-line treatment of metastatic nonsquamous NSCLC. N. Engl. J. Med..

[12] Davis AA, Patel VG (2019). The role of PD-L1 expression as a predictive biomarker: An analysis of all US Food and Drug Administration (FDA) approvals of immune checkpoint inhibitors. J. Immunother. Cancer.

[13] Khunger M (2017). Programmed Cell Death 1 (PD-1) Ligand (PD-L1) Expression in solid tumors as a predictive biomarker of benefit from PD-1/PD-L1 axis inhibitors: A systematic review and meta-analysis. JCO Precis. Oncol..

[14] Juneja VR (2017). PD-L1 on tumor cells is sufficient for immune evasion in immunogenic tumors and inhibits CD8 T cell cytotoxicity. J. Exp. Med..

[15] Taube JM (2014). Association of PD-1, PD-1 ligands, and other features of the tumor immune microenvironment with response to anti-PD-1 therapy. Clin. Cancer Res..

[16] Reuben A (2017). Genomic and immune heterogeneity are associated with differential responses to therapy in melanoma. NPJ Genom. Med..

[17] Jia Q (2018). Local mutational diversity drives intratumoral immune heterogeneity in non-small cell lung cancer. Nat. Commun..

[18] Munari E (2017). PD-L1 expression heterogeneity in non-small cell lung cancer: Evaluation of small biopsies reliability. Oncotarget.

[19] Gibney GT, Weiner LM, Atkins MB (2016). Predictive biomarkers for checkpoint inhibitor-based immunotherapy. Lancet Oncol..

[20] Huang AC (2017). T-cell invigoration to tumour burden ratio associated with anti-PD-1 response. Nature.

[21] Iwahori K (2019). Peripheral T cell cytotoxicity predicts T cell function in the tumor microenvironment. Sci. Rep..

[22] Miyara M (2009). Functional delineation and differentiation dynamics of human CD4+ T cells expressing the FoxP3 transcription factor. Immunity.

[23] Cyriac G, Gandhi L (2018). Emerging biomarkers for immune checkpoint inhibition in lung cancer. Semin. Cancer Biol..

[24] Fassler M (2019). Antibodies as biomarker candidates for response and survival to checkpoint inhibitors in melanoma patients. J. Immunother. Cancer.

[25] Teng F, Meng X, Kong L, Yu J (2018). Progress and challenges of predictive biomarkers of anti PD-1/PD-L1 immunotherapy: A systematic review. Cancer Lett..

[26] Arrieta O (2017). Expression of PD-1/PD-L1 and PD-L2 in peripheral T-cells from non-small cell lung cancer patients. Oncotarget.

[27] Jacquelot N (2017). Predictors of responses to immune checkpoint blockade in advanced melanoma. Nat. Commun..

[28] Jacquelot N, Zitvogel L, Eggermont AM (2018). Reply to ‘Challenging PD-L1 expressing cytotoxic T cells as a predictor for response to immunotherapy in melanoma’. Nat. Commun..

[29] Meniawy TM, Lake RA, McDonnell AM, Millward MJ, Nowak AK (2016). PD-L1 on peripheral blood T lymphocytes is prognostic in patients with non-small cell lung cancer (NSCLC) treated with EGFR inhibitors. Lung Cancer.

[30] Butte MJ, Keir ME, Phamduy TB, Sharpe AH, Freeman GJ (2007). Programmed death-1 ligand 1 interacts specifically with the B7–1 costimulatory molecule to inhibit T cell responses. Immunity.

[31] Schildberg FA, Klein SR, Freeman GJ, Sharpe AH (2016). Coinhibitory pathways in the B7-CD28 ligand-receptor family. Immunity.

[32] Brahmer JR (2010). Phase I study of single-agent anti-programmed death-1 (MDX-1106) in refractory solid tumors: Safety, clinical activity, pharmacodynamics, and immunologic correlates. J. Clin. Oncol..

[33] Topalian SL (2012). Safety, activity, and immune correlates of anti-PD-1 antibody in cancer. N. Engl. J. Med..

[34] Kim KH (2019). The first-week proliferative response of peripheral blood PD-1(+)CD8(+) T cells predicts the response to anti-PD-1 therapy in solid tumors. Clin. Cancer Res..

[35] Herbst RS (2014). Predictive correlates of response to the anti-PD-L1 antibody MPDL3280A in cancer patients. Nature.

[36] Postow MA (2016). Peripheral blood T cell subset phenotype analysis in melanoma patients treated with combination nivolumab + ipilimumab compared to ipilimumab alone. J. Clin. Oncol..

[37] Chen H (2009). Anti-CTLA-4 therapy results in higher CD4+ICOShi T cell frequency and IFN-gamma levels in both nonmalignant and malignant prostate tissues. Proc. Natl. Acad. Sci. U.S.A..

[38] Liakou CI (2008). CTLA-4 blockade increases IFNgamma-producing CD4+ICOShi cells to shift the ratio of effector to regulatory T cells in cancer patients. Proc. Natl. Acad. Sci. U.S.A..

[39] Wei SC (2017). Distinct cellular mechanisms underlie anti-CTLA-4 and anti-PD-1 checkpoint blockade. Cell.

[40] *FlowJo Software for Mac v. 10.4* (Becton, Dickinson and Company, 2019).

[41] *R: A Language and Environment for Statistical Computing* (R Foundation for Statistical Computing, 2013). https://www.r-project.org/. Accessed 29 Aug 2018.

[42] *A Package for Survival Analysis in R. v. R Package Version 2.38* (2015). https://CRAN.R-project.org/package=survival. Accessed 29 Aug 2018.

