# Supplementary material for: Changes in expression of PD-L1 on peripheral T cells in patients with melanoma and lung cancer treated with PD-1 inhibitors
Source: Sci Rep. 2021 Jul 28;11:15312. doi: 10.1038/s41598-021-93479-z (PMC8319434; doi:10.1038/s41598-021-93479-z)
Supplement: Supplementary file 1 — Supplementary Information. [file 41598_2021_93479_MOESM1_ESM.pdf]

Changes in expression of PD-L1 on peripheral T cells in patients with melanoma and lung cancer treated with PD-1 inhibitors

Sarah J. Dart^[1,2], Alistair M. Cook^[1,2], Michael J. Millward[1,3], Alison M. McDonnell[1,2], Wee L. Chin[1,2,3], Muhammad U. Hakeem[3], Tarek M. Meniawy[#1,3], Samantha E. Bowyer*[#1,3]

Affiliations:

[1]Faculty of Health and Medical Sciences, The University of Western Australia, Perth, Western Australia, Australia

[2]National Centre for Asbestos Related Diseases, Perth, Western Australia, Australia

[3]Department of Medical Oncology, Sir Charles Gairdner Hospital, Perth, Western Australia, Australia

***Corresponding author:** Dr Samantha Bowyer

Department of Medical Oncology, Sir Charles Gairdner Hospital, Hospital Avenue, Nedlands, Western Australia, Australia 6009

+61 8 6383 3000

samantha.bowyer@health.wa.gov.au

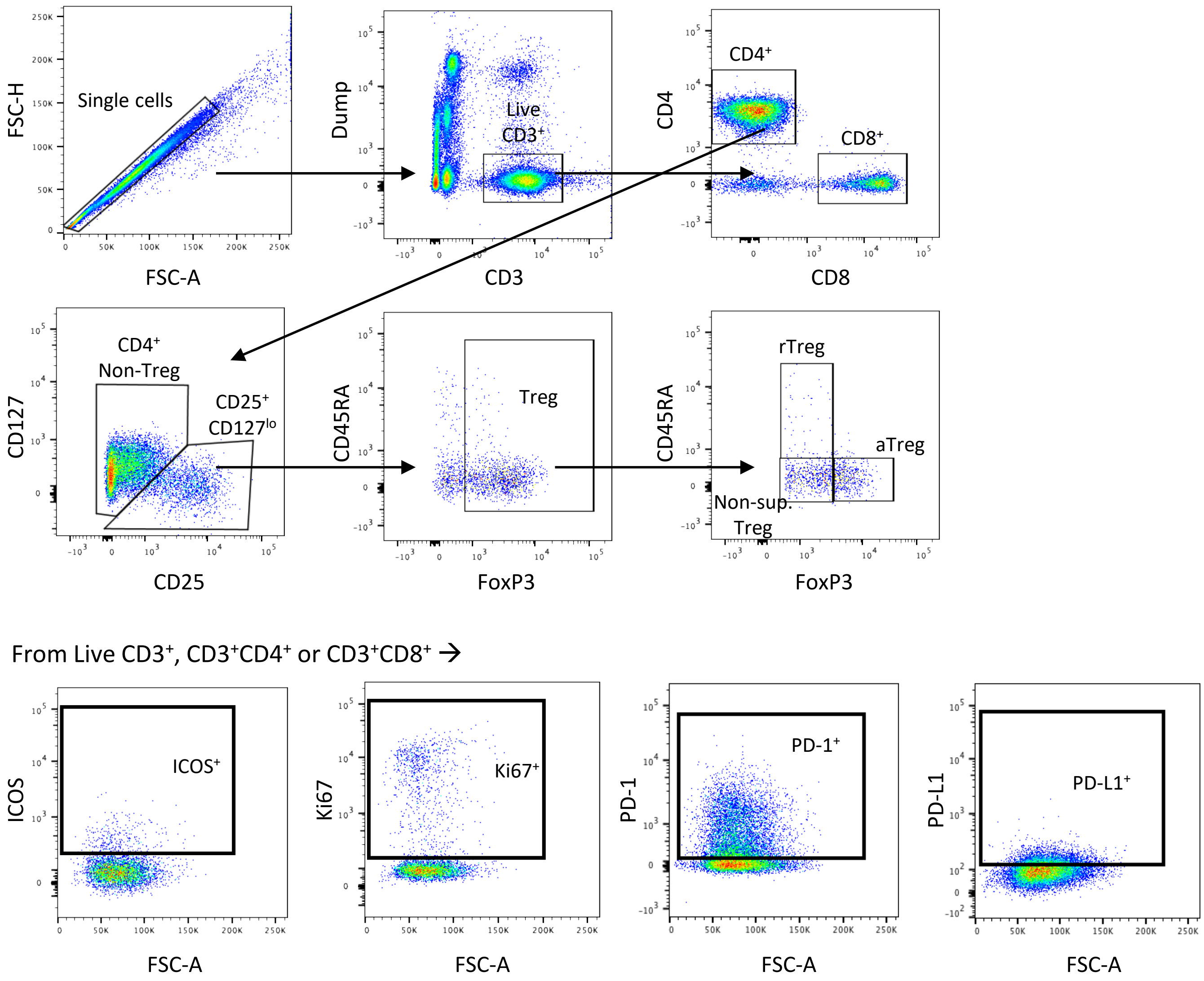

Supplementary Figure S1: Flow cytometry gating strategy.
Peripheral blood mononuclear cells were gated for single cells, dump$^-$ (CD19$^-$CD14$^-$live) CD3$^+$ cells, and then T cell subsets; CD8$^+$, CD4$^+$ Non-Treg, Treg, and Treg subsets. Expression of ICOS, Ki67, PD-1 or PD-L1 was assessed, with gates determined using fluorescence minus one controls.

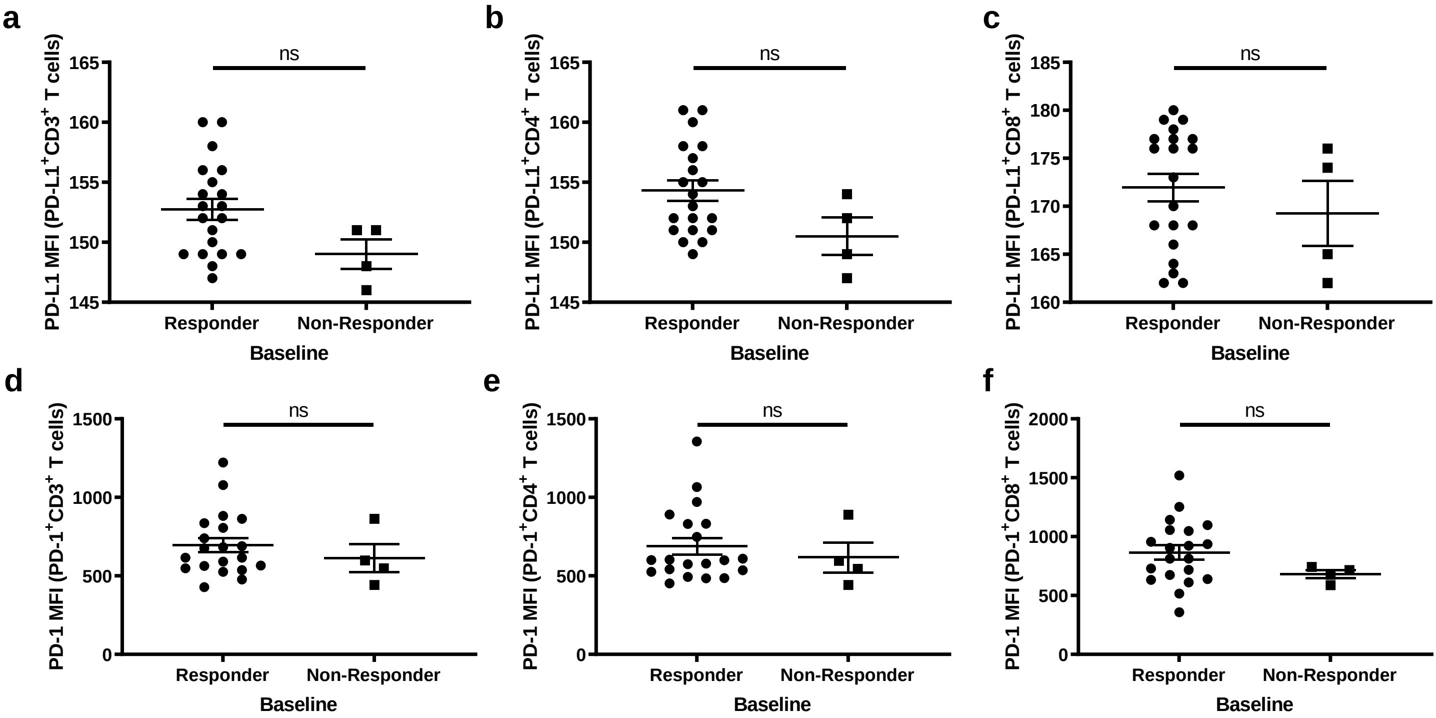

Supplementary Figure S2: Median Fluorescence Intensity of PD-L1 and PD-1 on T cells.
PBMC collected from patients were assessed for PD-L1 and PD-1 expression in the total $CD3^+$, $CD3^+CD4^+$ and $CD3^+CD8^+$ lymphocyte populations by flow cytometry, using fluorescence-minus one controls to set gates. Median Fluorescence Intensity (MFI) of PD-L1$^+$ (a-c) and PD-1$^+$ (d-f) cells of each population for responder (n=20) and non-responder patients (n=4) were compared using a Mann-Whitney test; error bars, SEM. See also Figure 2.

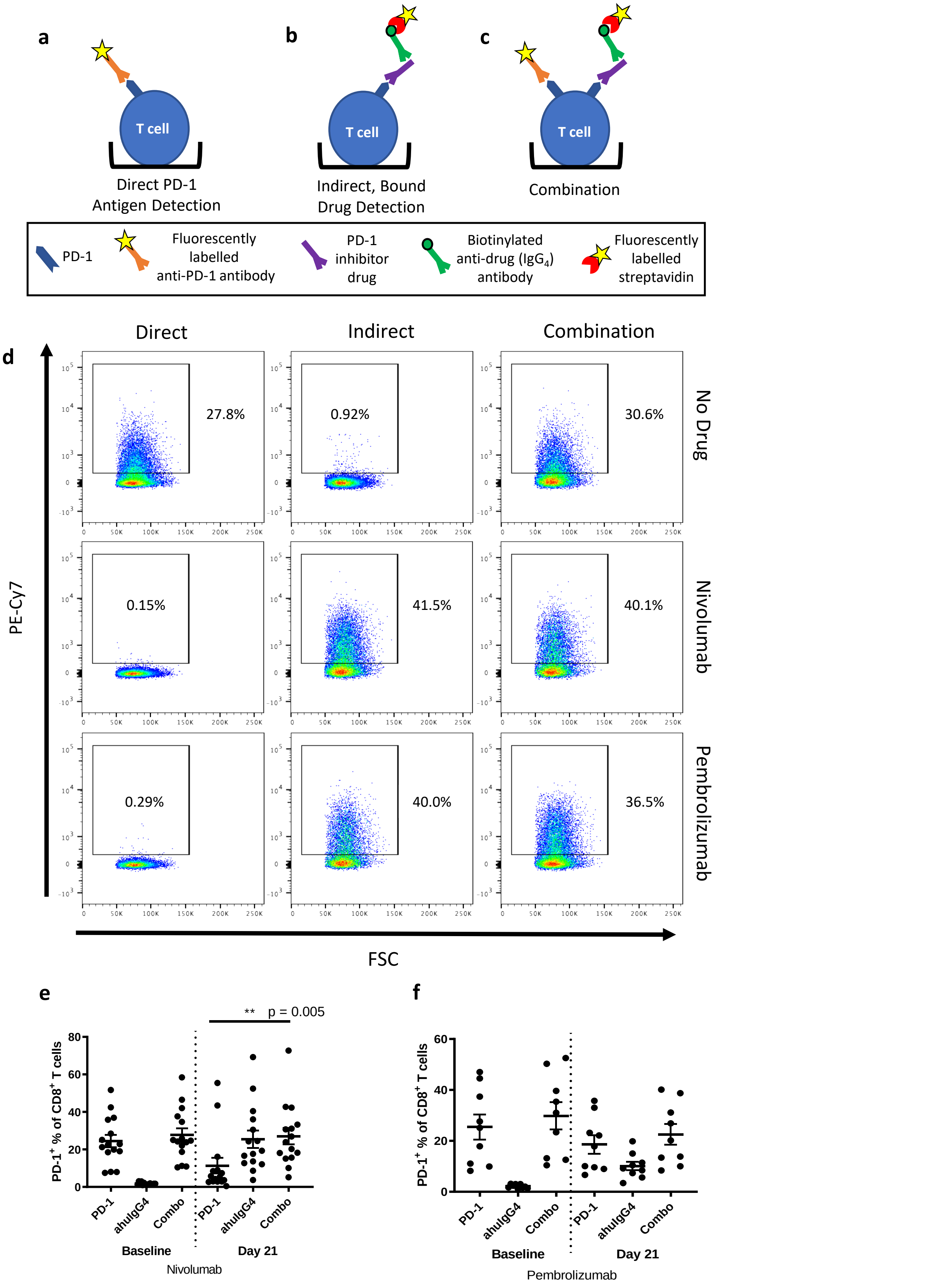

Supplementary Figure S3. Detection of PD-1 antigen expression on lymphocytes following treatment with anti-PD-1 therapy requires a two-pronged detection approach. Free PD-1 antigen was detected using classical flow cytometry staining (a), whilst drug-bound PD-1 was detected using a biotinylated anti-human IgG_4 (anti-drug) antibody followed by a fluorescently labelled streptavidin antibody (b). Using a combination of both detection methods allowed for all PD-1 antigen to be detected (c). Healthy human PBMC incubated with saturating concentrations of nivolumab or pembrolizumab were used as controls (d). PBMC of patients treated with (e) nivolumab (n=9) or (f) pembrolizumab (n=15) were assessed for proportions of PD-1 antigen expression using direct (PD-1), indirect (ahuIgG4) or combination (combo) methods as described, before (baseline) and after one cycle of treatment (day 21), Wilcoxon test; ** = p ≤0.005; error bars, SEM.
